# Assessment of Unsuspected Exposure to Drugs of Abuse in Children from a Mediterranean City by Hair Testing

**DOI:** 10.3390/ijerph110202288

**Published:** 2014-02-21

**Authors:** Simona Pichini, Oscar Garcia-Algar, Airam Alvarez, Massimo Gottardi, Emilia Marchei, Fiorenza Svaizer, Manuela Pellegrini, Maria Concetta Rotolo, Roberta Pacifici

**Affiliations:** 1 Drug Abuse and Doping Unit, Department of Therapeutic Research and Medicines Evaluation National Institute of Health, Roma 00161, Italy; E-Mails: emilia.marchei@iss.it (E.M.); manuela.pellegrini@iss.it (M.P.); mariaconcetta.rotolo@iss.it (M.C.R.); roberta.pacifici@iss.it (R.P.); 2 Unitat de Recerca Infància i Entorn (URIE), Paediatric Service, Institut Hospital del Mar d’Investigacions Mèdiques (IMIM), Barcelona 08003, Spain; E-Mails: 90458@hospitaldelmar.cat (O.G.-A.); 60809@hospitaldelmar.cat (A.A.); 3 Laboratorio di Sanità Pubblica (LSP), Azienda Provinciale Servizi Sanitari, Trento 38010, Italy; E-Mails: massimo.gottardi@comedicaltrento.it (M.G.); fiorenza.svaizer@apss.tn.it (F.S.)

**Keywords:** hair testing, drugs of abuse, children, unsuspected exposure

## Abstract

Hair testing was used to investigate the prevalence of unsuspected exposure to drugs of abuse in a group of children presenting to an urban paediatric emergency department without suggestive signs or symptoms. Hair samples were obtained from 114 children between 24 months and 10 years of age attending the emergency room of Hospital del Mar in Barcelona, Spain. Hair samples from the accompanying parent were also collected. The samples were analyzed for the presence of opiates, cocaine, amphetamines, and cannabinoids by ultra-performance liquid chromatography-tandem mass spectrometry. Parental sociodemographics and possible drug of abuse history were recorded. Hair samples from twenty-three children (20.1%) were positive for cocaine (concentration range 0.15–3.81 ng/mg hair), those of thirteen children (11.4%) to cannabinoids (Δ9-THC concentration range 0.05–0.54 ng/mg hair), with four samples positive to codeine (0.1–0.25 ng/mg hair), one positive for 2.09 ng methadone per mg hair and one to 6-MAM (0.42 ng/mg hair) and morphine (0. 15 ng/mg hair) . In 69.5 and 69.2% of the positive cocaine and cannabinoids cases respectively, drugs was also found in the hair of accompanying parent. Parental sociodemographics were not associated with children exposure to drugs of abuse. However, the behavioural patterns with potential harmful effects for the child’s health (e.g., tobacco smoking, cannabis, benzodiazepines and/or antidepressants use) were significantly higher in the parents of exposed children. In the light of the obtained results (28% overall children exposure to drugs of abuse) and in agreement with 2009 unsuspected 23% cocaine exposure in pre-school children from the same hospital, we support general hair screening to disclose exposure to drugs of abuse in children from risky environments to provide the basis for specific social and health interventions.

## 1. Introduction

Drug testing in hair is a unique analysis in pharmacotoxicology for establishing a history of consumption or passive exposure for up to several months disclosing a possible chronic active or passive intake of an abused drug [1]. Apart from known application in forensic toxicology, drug testing in hair suitably fit for proposes of epidemiological studies to objectively assess repetitive exposure to a certain drug in specific populations (e.g., general population pregnant women, newborns, children) [2,3,4,5,6]. Specifically, in case of newborns and children, the non invasiveness of matrix collection rendered hair testing the best choice to disclose past recurring exposure to drugs of abuse [7,8,9].

Compared to data from other European Union member states, in 2011 Spain showed the highest percentage of cocaine and cannabis consumption: 2.3 and 10.2% annual prevalence in the general population (15–64 years) with 30% consumption of both drugs and cocaine use levels exceeding even those reported from America [10,11]. Conversely, amphetamines and heroin consumption significantly decreased to a 0.7 and 0.1% annual consumption in the general population [11].

This alarming consumption explained prenatal exposure figures of 2.6, 4.7 and 5.3% to cocaine, heroin and cannabis in newborns from a Barcelona (Spain) cohort in 2004 [12,13] and in 2009 resulted in repeated exposure to cocaine in 23.3% infants and pre-school children living in a domestic environment of drug abusers [9].

Concerns regarding the above reported results and the health effects of repeated exposure to drugs of abuse in childhood together with the stabilization of high levels of drug abuse in Spain prompted some investigators to investigate by hair testing the prevalence of unsuspected exposure to principal drugs of abuse in children without signs or symptoms suggestive of exposure, attending the paediatric emergency department of the Hospital del Mar in Barcelona, during a two month period. A highly sensitive and specific analysis of drugs of abuse in hair by ultra-performance liquid chromatography tandem mass spectrometry (UPLC-MS/MS), recently developed by our investigation group was applied in order to disclose also minute amounts of drugs and eventual metabolite in children hair suggestive of any exposure to illicit drugs [14].

## 2. Methods

### 2.1. Subjects and Samples

This study was conducted in the Paediatric Emergency Department of the Hospital del Mar of Barcelona, Spain, between March and April 2013. The hospital is located in an urban area with low socioeconomic status and a high percentage (more than 40%) of immigrants [9,12]. Hair samples, cut at the scalp, were collected from all the children between 24 months and 11 years of age, admitted to the Emergency Department for a variety of general medical complaints. Since hair grows at a rate of approximately 1 cm/month, in order to document a possible repeated exposure, a minimum of 4 cm hair was required. Children younger than 24 months were excluded to avoid the possibility that a positive result of any drug of abuse in hair samples could be partly due to prenatal exposure. When children attending emergency rooms were accompanied by one or two of their parents, they were asked to donate hair samples, as well. The study was approved by the Institutional Ethics Committee (CEIC-IMAS, project number 1333/2012), conducted in accordance with the Declaration of Helsinki; and signed informed consent was obtained from the accompanying parent. Parental sociodemographics and possible toxic habits were recorded using standard structured questionnaire usually applied at the hospital and in our previous investigations [9,12,13]. Results from the clinical examination of the children were available from medical records.

### 2.2. Hair Samples Analysis

The hair samples from adults were firstly screened for the presence of drugs of abuse by immunometric analysis [14] and positive samples confirmed by subsequent confirmatory UPLC-MS/MS methodology as reported elsewhere [14]. It was decided to screen only the adult hair samples, since the amount of children’ samples in many cases (samples with less than 30 mg hair) did not allow both screening and confirmation assay. Adult samples were considered positive or negative on the basis of cut-offs for screening and confirmation techniques recommended by the Society of hair testing [15]. Conversely, in case of paediatric samples, they were considered positive when analyte concentration was equal of higher than the confirmatory analysis limit of quantification (LOQ). Specifically, LOQ was 0.1ng/mg hair for opiates and cocaine and 0.05 ng/mg hair for benzoylecgonine and cannabinoids. 

### 2.3. Statistical Analysis

Statistical analysis for the continuous variables was done with the Student’s t test for two groups of data and the analysis of variance (ANOVA) for three or more groups of data. The Fisher’s exact test was used for the comparison of categorical data. For the analysis of individual percentages of exposed *vs*. unexposed children, the binomial test was used. Statistical significance was set at *p* < 0.05.

## 3. Results

Finally, 228 (114 parental—17.5% fathers—and 114 paediatric) samples were collected and the proximal hair segment of 4 cm was finally analyzed since this length was available for the totality of specimens.

Immunometric screening of adult hair samples gave three positive results for opiates class, 19 positives for cocaine class, three for methadone, 18 for cannabinoids class. UPLC-MS/MS analysis confirmed all the cases positive to screening test identifying: six monoacetylmorphine 6-MAM (3.43, 1.60 and 0.90 ng/mg hair), morphine (1.34, 0.83 and 0.45 ng/mg hair) and codeine (0.32, 0.30 and 0.30 ng/mg hair) in the three samples positive to opiates class; cocaine (median value: 2.05 ng/mg hair) and benzoylecgonine (median value: 1.25 ng/mg hair) in the 19 positives samples; methadone (2.68, 2.02 and 1.01 ng/mg hair) in the samples positive to methadone class and finally Δ9-THC (median value: 1.36 ng/mg hair), cannabinol (median value: 0.14 ng/mg hair) and cannabidiol (median value: 1.25 ng/mg hair) in the 18 samples positive to cannabinoids class (Table 1).

**Table 1:** Drugs of abuse content in adult hair samples.

Sample ID	Opiates (ng/mg hair)				Cocaine (ng/mg)		Cannabinoids (ng/mg)		
	6-MAM	Morphine	Codeine	Methadone	Cocaine	Benzoylecgonine	Δ-9 THC	CBD	CBN
M4							1.50	1.12	0.11
D7							1.35	1.26	0.24
D11					1.23	0.63			
M18							2.43	1.66	0.63
M20					1.46	0.66	1.39	1.20	0.09
M21					1.44	0.34	2.10	1.11	0.12
M23							1.19	1.12	0.14
M27					0.71	4.67			
M32	3.43	1.34	0.32						
D34							3.25	1.25	0.05
D39							1.35	1.17	0.05
M42							3.11	2.01	1.01
M43					2.36	0.69			
M48					1.35	1.51			
M55							1.27	1.14	0.14
D61				0.41	2.05	0.95	1.36	1.23	0.21
M65							1.30	1.38	0.66
M72					1.45	0.73			
M75					4.66	1.54			
M76					1.96	1.41			
M81					2.09	0.84			
M83					4.53	3.76			
M85					2.06	1.78			
M89	1.60	0.83	0.30						
M92					1.47	0.72	2.45	1.26	0.09
M93					1.85	1.44			
M96					1.33	1.52			
M97					3.41	1.54			
M102							1.32	1.25	0.12
M105					2.23	1.25			
M106	0.90	0.45	0.30	0.32					
M113							1.19	1.25	0.05
M118							1.23	1.63	0.63
M121							1.36	1.27	0.30
D123					4.73	2.14	2.03	1.18	0.18
M124				0.31					

In case of children hair samples, UPLC-MS/MS analysis disclosed: one sample positive to 6-MAM (0.42 ng/mg hair) and morphine (0.15 ng/mg hair), four samples positive to codeine (median value 0.17 ng/mg hair) 23 samples positive to cocaine (median value: 0.54 ng/mg hair) and benzoylecgonine (median value: 0.18 ng/mg hair), one sample positive to methadone (2.09 ng/mg hair) and finally 13 samples positive to -9 THC (median value: 0.16 ng/mg hair) and cannabidiol (median value: 0.15 ng/mg hair) with just one sample also positive for cannabinol (0.79 ng/mg hair) (Table 2). Age mean and median of children positive to repeated exposure to drugs of abuse were 5.9 and 5 years, respectively.

**Table 2:** Drugs of abuse content in children hair samples.

Sample ID	Opiates (ng/mg)				Cocaine (ng/mg)		Cannabinoids (ng/mg)		
	6-MAM	Morphine	Codeine	Methadone	Cocaine	Benzoylecgonine	Δ-9 THC	CBD	CBN
C5							0.05	0.05	
C7							0.07	0.08	
C8					0.58	0.27	0.21	0.15	
C18					0.51	0.45	0.05	0.05	
C20					0.17	0.08	0.05	0.05	
C21					0.16	0.07	0.18	0.09	
C23					0.15	0.07			
C27					0.38	0.37			
C30					0.68	0.25			
C35			0.12						
C42							0.22	0.20	
C43					0.15	0.08			
C48					0.54	0.12			
C61				2.09	0.17	0.19	0.20	0.16	
C65							0.16	0.15	
C72					0.56	0.20			
C74					0.15	0.15			
C75					0.54	0.33	0.09	0.05	
C76					1.65	0.40			
C79			0.25						
C83					0.15	0.10			
C87					0.62	0.08			
C89	0.42	0.15							
C92					0.74	0.21			
C93					0.53	0.18	0.29	0.23	
C96					0.58	0.09			
C97					0.45	0.11			
C98			0.11						
C105			0.23		1.31	0.24			
C116					0.78	0.07			
C118							0.54	0.41	0.79
C123					3.81	0.55	0.12	0.15	

Positivity rate of children hair samples for the different drugs match matched to parental positivity rate in one case out of three for 6-MAM and morphine, in the single case positive to methadone, in 69.5% cases for cocaine and 69.2 % cases for cannabinoids. Parental ethnicity was not associated with drugs use and subsequent children exposure nor was maternal or paternal socioeconomic status. Conversely, children exposure to drugs of abuse was associated with a significantly higher maternal smoking habit and greater number of cigarettes smoked by both parents, a higher percentage of cannabis consumption by both parents, and higher maternal benzodiazepines and/or antidepressants use (Table 3). Interestingly, none of the parents declared any consumption of cocaine or opiates at any time in their life.

**Table 3:** Self declared parental sociodemographics and toxic habits for the 114 studied children according to hair analysis.

	Children not exposed to any drug of abuse (*n* = 82)	Children exposed to any drug of abuse (*n* = 32)
**Parental socio-demographics**		
**Mother’s nationality (%)**		
Spanish	57.5	62
Non Spanish	42.5	38
**Father’s nationality (%)**		
Spanish	60.2	65.4
Non Spanish	39.8	34.6
**Employed mother** (Yes/No) **(%)**		
No	32.4	35
**Mother’s profession (%)**		
Managerial, Professional & Skilled (non-manual)	8.5	7.7
Skilled (manual) & Partly skilled	40.3	42.3
Unskilled	51.2	50
**Employed Father** (Yes/No) **(%)**		
No	2.3	3.4
**Father’s profession (%)**		
Managerial, Professional & Skilled (non-manual)	10.6	10.5
Skilled (manual) & Partly skilled	25.9	30.8
Unskilled	63.5	58.7
**Parental drug consumption at the visit**		
**Tobacco Smoking**		
Mother (%)	27.2	49.6 **
Number of daily cigarettes, mean (S.D.)	5.4 (3.8)	13.8 (3.5) **
Father (%)	41.7	49.1
Number of daily cigarettes, mean (S.D.)	8.8 (3.3)	16.7 (6.5) **
**Alcohol consumption**		
Mother (%)	29.5	29.3
Father (%)	39.3	43.9
**Cannabis Use**		
Mother (%)	6.8	21.6 **
Father (%)	10.2	31.2 **
**Opiates and cocaine use**		
Mother (%)	0	0
Use during pregnancy (%)	0	0
Father (%)	0	0
**Benzodiazepines or Antidepressant use**		
Mother (%)	7.8	27.4 **
Father (%)	4.7	9.8

** *p* > 0.05.

## 4. Discussion

Our study highlighted an extremely high incidence of unsuspected exposure to drugs of abuse by hair testing in children from a low socioeconomic status cohort in a Mediterranean city [16]. Hair testing for opiates, cocaine, amphetamines, methylendioxyderivatives, cannabinoids and methadone was provided using a highly specific and sensitive screening and confirmation methodology recently developed coupling an immunoassay specific for keratin matrix with last generation ultra-performance liquid chromatography tandem mass spectrometry [14]. In case of parental hair samples, the cut-offs recommended by the Society of Hair Testing were used to discriminate positive samples for both screening and confirmation analyses [15]. Using these cut-offs, no false positive and negative results were found applying screening analysis to adult hair samples, which were always confirmed by chromatographic/mass spectrometric assay. In case of children hair samples, results were considered positive not on the basis of recommended cut-offs—which indeed do not exist for paediatric samples—but when analyte concentration was equal of higher than the confirmatory analysis limit of quantification. The reason was that the international cut-offs for positivity to drugs of abuse were established considering values for adult population of consumers and non consumers of those substances. Conversely, in case of children we considered that none of them could have been assimilated to a consumer (even if accidental intake could not be excluded) since they were younger than 10 years of age, nor intrauterine exposure could have been occurred since they were older than 2 years of age and examined hair strand was of 4 cm, corresponding to the previous 4–5 months. Furthermore, the last generation UPLC-MS/MS allowed a greater sensitivity which permitted to disclose the maximum possible number of children exposed to drugs of abuse, including cannabinoids, whose hair concentration is extremely low even in active consumers [1].

Paediatric hair testing in this study confirmed that children positivity rate to repeated exposure to drugs of abuse matched to parental positivity rate to the same drug in the paediatric sample positive for 6-MAM and morphine, in the single case positive to methadone, in 69.5% cases positive to cocaine and 69.2% cases to cannabinoids, confirming that the first source of paediatric exposure to drugs of abuse are the parents and more in general a risky parental environment. Indeed, in case of children with hair samples positive to drugs of abuse, whose accompanying parent was negative, it cannot be excluded exposure to the other parent or care-giver consumption of drugs or contact with contaminated surroundings. Conversely, the four paediatric cases positive to codeine could have been attributed to the use of antitussive syrups, which in any case was not declared at the visit interview.

The results of the present study are in complete agreement with a previous study of 2009 concerning unsuspected exposure to cocaine in preschool children (younger than 5 years of age) attending the same Emergency Department at Hospital del Mar of Barcelona. That time, we found a 23.3% preschool children (1–5 years of age) positive for cocaine (concentration range 0.3–5.96 ng/mg of hair) with one sample also positive for MDMA and another for opiates. In 88% of the positive cases, cocaine was also found in the hair of the accompanying parent. In that study too, parental sociodemographics were not associated with children’s exposure to cocaine, nor somatometry of children at birth. However, the behavioural patterns with potential harmful effects for the child’s health (e.g., tobacco smoking, cannabis, benzodiazepines and/or antidepressants use, shorter breastfeeding time) were significantly higher in the parents of exposed children [9]. We attributed the high percentage of positive cases to the fact that the preschool children stay at home with a parent or a caregiver the majority of time, but the present study show that this was not the case since high percentages of paediatric exposure was not only found in preschool children, but also in case of 2–11 years old children. It’s noteworthy that in 2009 prevalence of cocaine consumption in the general population was around 3%, while in 2011 it decreased to 2.3%. Nonetheless, this decrease is attributable to all the Spanish population, and not to the specific Barcelona area investigated in our study, where consumption and consequent pediatric exposure has been shown to be higher than the average country data [7,8]. Interestingly, in the same environment, in 2005, we reported a hidden prenatal chronic exposure to cocaine in 4.4% newborns, together with 5.3 and 8.7% exposure to cannabis and opiates, respectively [12]. Taken together, these two outcomes demonstrate the magnitude of the problem of exposure to drugs of abuse among children in this environment. 

Concerning exposure to drugs of abuse in paediatric population of this study, it should be said that: firstly external contamination have to be excluded since and extended standardized hair washing was included in the procedure for hair testing. Secondly, as reported by international literature, even if the route(s) of children “passive or active” exposure to drugs of abuse- (e.g., parental consumption in the household, contact with drugs-contaminated surroundings, crawling and putting contaminated objects in the mouth, accidental ingestion), cannot be easily established, contact occurred as proved by positive hair testing with possible risk of subsequent severe intoxications [17,18].

While it has been proved that acute exposure to cocaine during childhood is associated with neurologic manifestations such as focal and generalized seizures in children eight years of age and younger and alterations in mental status, including delirium stupor and coma in older children [19], and even death in cases of oral or inhaled routes of intoxication [8], the long term effects of a repeated postnatal exposure to cocaine have not been clarified yet.

Similarly, whereas children exposure to cannabis was reported to cause developmental delay, hyperactivity, lethargy ataxia, and respiratory insufficiency [20,21], no information on the effects of pediatric chronic exposure to that drug is known.

Nonetheless, even if it is difficult to identify a core set of physical, cognitive, or behavioural problems that can be directly attributed to postnatal (or pre and postnatal) exposure to drugs of abuse, it is well known that paediatric exposure to drugs of abuse is frequently associated with poor child care by the care giver.

In this concern, our previous study showed that parents of cocaine exposed children present, to a significantly higher extent, behavioural patterns with potential harmful effects for the child’s health (e.g., tobacco smoking, cannabis use, benzodiazepines and/or antidepressant use, shorter breastfeeding time). Indeed, a significantly high percentage of those children (11.8%) showed a failure to thrive which would require nutritional assessment and follow-up [9].

The parental risky behaviours are confirmed in the present study: mothers and fathers of children exposed to drugs of abuse smoke more cigarettes and cannabis joints and consume more benzodiazepines and antidepressant drugs.

These confirmed occurrences let us hypothesizes that these children could receive poor familiar attention, in a country like Spain, where drug exposed children are not protected by any specific law promoting foster care or programmed social intervention. Also, consumer parents are not aware of risk of passive environmental exposure in children living in the same environment.

## 5. Conclusions

In conclusion, our present study corroborates the already observed alarming prevalence of unsuspected exposure to drugs of abuse in children from low socioeconomic environment in a Mediterranean city. The obtained information can be taken into account and translated in all the countries where a suspicion exists of paediatric exposure to drugs of abuse following parental consumption. Hair testing for drugs of abuse in paediatric population proved to be an essential assay to disclose a repeated exposure in children from risky environments, which might otherwise not be identified. 

Since hair testing for drugs of abuse is still an expensive test due to the required specific skills and instrumentation, unfortunately it cannot be proposed as a general screening for the paediatric population. As maximum cost benefit ratio, we advocate performing this test in the paediatric population attending emergency wards or general hospital for any complaint when:

- the hospital is located in a area of very high percentage of drug consumption and socioeconomic difficulties;
- there is clinical suspicion of acute intoxication from any possible drug in the child;
- there is a clinical suspicion of chronic intoxication from any possible drug in the child;
- there is a suspicion or evidence or declaration of drug addiction in the accompanying parent;
- there is evidence of a risky environment around the child.

Specific social and health interventions towards exposed children and their families may help in preventing and treating possible intellectual, emotional-behavioural and neurologic disorders in the exposed children. This strategy can also lead to educating and counselling parents on potentially deleterious effects of drugs of abuse available and consumed at home even if it is done not in the presence of the children.
